# In Situ Lubrication in Forging of Pure Titanium Using Carbon Supersaturated Die Materials

**DOI:** 10.3390/nano14040363

**Published:** 2024-02-15

**Authors:** Tatsuhiko Aizawa, Tatsuya Funazuka, Tomomi Shiratori

**Affiliations:** 1 Surface Engineering Design Laboratory, Shibaura Institute of Technology, Tokyo 144-0045, Japan; 2 Engineering School, Toyama University, Toyama 930-8555, Japan; funazuka@eng.u-toyama.ac.jp (T.F.); shira@eng.u-toyama.ac.jp (T.S.)

**Keywords:** carbon supersaturation, nanostructures, SKD11 die, βSiC coating die, in situ solid lubrication, low friction, galling-free forging

## Abstract

A new solid lubrication method was proposed for dry forging of pure titanium with high reduction in thickness. A free-carbon tribofilm was formed in situ at the hot spots on the contact interface to protect the die surfaces from severe adhesion of work materials. This film consisted of the free carbon, which isolated from the carbon supersaturated die substrate materials, diffused to the contact interface and agglomerated to a thin film. Two different routes of carbon supersaturation process were developed to prepare carbon supersaturated ceramic and metal dies for the dry forging of pure titanium wires. A pure titanium bar was utilized as an easy-to-adherent work material for upsetting in dry and cold. The round bar was upset up to 70% in reduction in thickness with a low friction coefficient from 0.05 to 0.1 in a single stroke. Work hardening was suppressed by this low friction. SEM-EDX, EBSD and Raman spectroscopy were utilized to analyze the contact interface and to understand the role of in situ formed free-carbon films on the low friction and low work hardening during forging. Precise nanostructure analyses were utilized to describe low friction forging behavior commonly observed in these two processes. The in situ solid lubrication mechanism is discussed based on the equivalence between the nitrogen and carbon supersaturation processes.

## 1. Introduction

**Figure 1:**
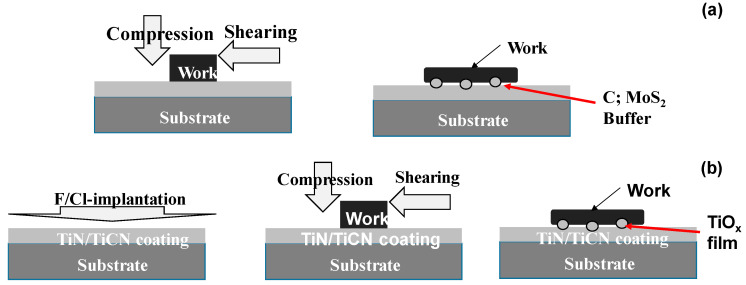
A schematic view of two types of solid lubrication mechanisms for dry metal forming and machining. (**a**) Solid lubricating coatings with molybdenum di-sulfide (MoS_2_) and carbon (C) to form the MoS_2_ or carbon agglomerates in situ, isolated from coating during compression and sliding [5,6], and (**b**) in situ lubrication by the synthesized tribofilms of intermediate titanium oxide (TiO_x_; 1 < x < 2) from the F/Cl implanted TiN/TiCN coating [7,8].

Minimum quantity lubrication (MQL) with less use of lubricating oils [1] and dry lubrication with positive use of solid lubricating mechanisms [2] have been highlighted to reduce the environmental burdens in practical operations toward green metal forming [3]. In particular, solid lubrication is indispensable in dry metal forming and dry machining to reduce tool wear and to qualify product surfaces [4]. The use of solid lubricants such as graphite and MoS_2_ (Molybdenum die-sulfide) provides a way to form the slipping buffer layer between the die and work [5]. As depicted in Figure 1a, powders and coatings have been utilized as a lubricating buffer layer in practice. Self-lubricating coating provides a means to prevent the protective coating from oxidation wear and severe galling as depicted in Figure 1b [6,7,8]. When using the ta-DLC or tetragonal amorphous carbon coating, the in situ synthesized polymer layer is wrought as a layer for boundary lubrication [6]. In particular, the fluorine- and chlorine-bearing titanium nitride coating is wrought to reduce the friction and specific wear volume in dry forming [7]. These two categorized methods have been effective in most dry metal forming. However, severe galling wears cannot be avoided when using these approaches alone, especially when working with titanium and titanium alloys [8,9,10].

Most DLC coatings and binary/ternary nitride coatings are subjected to the adhesion of fresh titanium fragments, even in BOD (ball-on-disc) testing [8,9]. TiN, TiCN and TiAlN coatings experience high friction transients before seizure to titanium balls [8]. The textured substrate surface exhibits relatively low friction with μ = 0.2 under MoS_2_ lubrication [9]. Irrespective of die material selection for deep drawing pure titanium sheets, the drawing ratio was limited to be far less than 2.0 [10]. In every forging step, titanium oxide debris particles can splash in the air and be deposited onto the die surface in practical operation [11]. In the free-forging and near-net forging process in hot conditions [12], galling easily occurs in every step of the hot forging processes.

**Figure 2:**
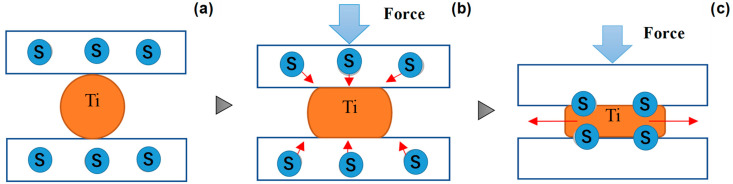
A schematic view of the in situ solid lubrication of the contact interface between die and work by tribofilm. (**a**) Supersaturating of interstitial solutes (S) into the punch and die, (**b**) isolating of interstitial solutes and its boundary/zone diffusing in the punch and die substrate, and (**c**) in situ agglomeration of interstitial solutes and formation of S-tribofilm on the contact interface.

Figure 2 depicts a new solid lubrication method with the use of in situ formed interstitial solute film on the contact interface. As depicted in Figure 2a, the solute atoms or clusters are embedded into the die substrate. Under the stress gradient across the contact interface between the die and metallic work, these solutes isolate from the die matrix, diffuse to the interface through the nanocluster boundary network in Figure 2b, and agglomerate by themselves to form a tribofilm on the interface in Figure 2c. In the literature, carbon has been employed as the interstitial solute (S). After [13], free carbon supersaturated AISI316 stainless steels with an extremely high content of 12 at% were achieved through gas-phase carburizing at 743 K. The carbon supersaturated (CS) steels can be prepared by surface treatment to drive the supersaturation process. In particular, free carbon supersaturated and clustered in steels as reported in [14]. Free carbon isolated from the CS state during steel processing as stated in [15]. These findings in the literature suggest that the in situ solid lubrication model in Figure 2 can be applied to steel dies by developing an adequate carbon supersaturation process.

In previous studies, low temperature plasma carburizing was utilized to facilitate carbon supersaturation into steel die substrates [16]. SKD11 and AISI420J2 dies were carburized at low holding temperatures to prepare the carbon supersaturated dies (CS dies) for cold upsetting of pure titanium works, respectively. As reported in [11,17], the reduction in thickness was limited by 20% by using the bare tool and stainless steel dies due to the risk of galling on the interface in normal forging operations. When using the CS-SKD11 and CS-AISI420J2 punches and dies, no adhesion of fresh titanium flakes and no deposition of titanium oxide debris were seen after forging even with a reduction in thickness of 70%.

In addition, the hot CVD (chemical vapor deposition) process was utilized to facilitate carbon supersaturation into the SiC die substrates by controlling the flow rate ratio of methane into source gas for the chemical deposition of β-SiC coating [18]. The thick CS-βSiC layer was coated onto the SiC die substrate with a thickness greater than 4 mm. Even in cold upsetting of pure titanium works, they were uniformly forged under a reduction in thickness of 70% without any adhesion of titanium flakes or debris particles. The 3C-structured β-SiC coating layer with its sufficiently high thickness was directly utilized as a die material for the forging process of titanium and titanium alloy works. This CS-βSiC die has high enough heat resistance to be used for hot and warm forging processes at elevated temperatures higher than 1000 K.

In the present paper, the in situ tribofilm formation model for solid lubrication is stated with reference to previous studies on the activity of interstitial solutes in steels even under the stress gradient. Two types of carbon supersaturation process are introduced to prepare the CS-SKD11 dies and CS-βSiC-coated SiC dies for experimental demonstration on the in situ solid lubrication model. The low temperature plasma carburizing system is utilized to achieve carbon supersaturation in SKD11 discs, punches, and dies, respectively. A thermal CVD system is also utilized to achieve carbon supersaturation in βSiC with its co-deposition onto the SiC dies by controlling the carbon source. The upsetting processes are performed to prove that the low friction and low galling-wear conditions are preserved by using CS tools. SEM (scanning electron microscopy)-EDX (energy dispersive X-ray spectroscopy) and Raman spectroscopic analyses are employed to describe the free-carbon tribofilm formation on the contact interface. The in situ solid model on the carbon solute isolation, diffusion and agglomeration is discussed through the precise nanostructure analysis of the CS tools based on the equivalence between the nitrogen and carbon supersaturation processes.

## 2. Materials and Methods

### 2.1. Two Carbon Supersaturation Processes

Two types of carbon supersaturation process were utilized to prepare the CS-metallic tools and the CS-ceramic tools for experimental demonstration on the in situ solid lubrication model. The low temperature plasma carburizing system (YS-Electric Industry, Co., Ltd.; Kofu, Japan) was used for carbon supersaturation into the heat-treated SKD11 disc and die substrates under the processing conditions in Table 1.

**Figure 3:**
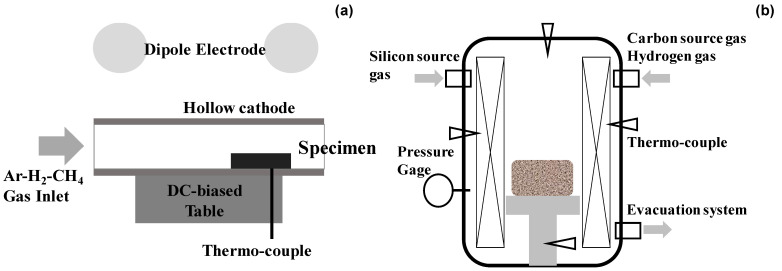
Schematic view of the two types of carbon supersaturation processes to die substrate. (**a**) Low temperature plasma carburizing into the metallic die substrate and (**b**) hot CVD process to control the carbon content in synthesis of 3C-structured βSiC coating on the SiC sintered substrate.

**Figure 4:**
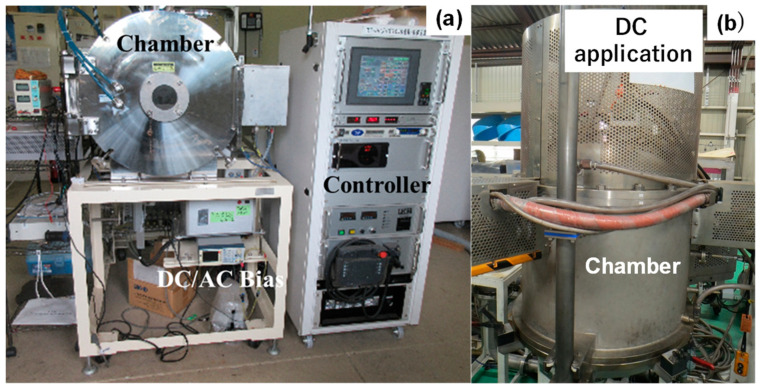
Two types of carbon supersaturation processes to die substrates. (**a**) Low temperature plasma carburizing system into the metallic die substrate and (**b**) hot CVD process to control the carbon content in synthesis of 3C-structured βSiC coating on the SiC sintered substrate.

As schematically depicted in Figure 3a, a hollow cathode setup was utilized to intensify the population of CH radicals and carbon ions. A thermocouple was embedded into this hollow unit in the vicinity of the substrates. In the following plasma carburizing process, the specimen was placed into the hollow cathode setup before evacuation down to the base pressure of 0.01 Pa. Argon gas was introduced at the specified pressure for heating up to 673 K. After presputtering for 1.8 ks, the specimen was carburized at 673 K for 14.4 ks. The overview of this carburizing system is depicted in Figure 4a. The matching between input and output powers was automatically adjusted by the frequency control around 2 MHz in an RF-power generator.

Thick SiC coatings were highlighted in the literature [19,20]. Among them, a thermal CVD system (Shinko-Seiki, Co., Ltd.; Kobe, Japan) was used to achieve carbon supersaturation to the co-deposited βSiC coating onto the SiC die substrate by controlling the carbon source gas flow rate. As illustrated in Figure 3b, a sintered SiC block with an average porosity of 0.5% was prepared as a substrate to make a thick SiC coating via the thermal CVD process at 1500 K for 108 ks. Silicon chloride and methane gases were selected as the silicon and carbon sources, respectively. They were mixed with hydrogen gas and introduced using a flow rate of 500 mL/min to the thermal reactor in the present CVD system. The thickness of the βSiC coating was 4 mm. After cooling down in an inert gas atmosphere, the βSiC-coated SiC block was directly cut using a diamond saw and polished using chemical buffing to shape the SiC-coated SiC punch and die. An overview of thermal CVD system is depicted in Figure 4b.

### 2.2. Work Materials

Pure titanium wires and plates were respectively utilized for the upsetting experiments. The chemical composition of pure titanium materials consists of hydrogen by 0.0012 mass%, oxygen by 0.097 mass%, nitrogen by 0.007 mass%, iron by 0.042 mass%, carbon by 0.007 mass%, and titanium for balance. The flow stress was 230 MPa, and the Young’s modulus was 110 GPa.

### 2.3. Upsetting Experiments

**Figure 5:**
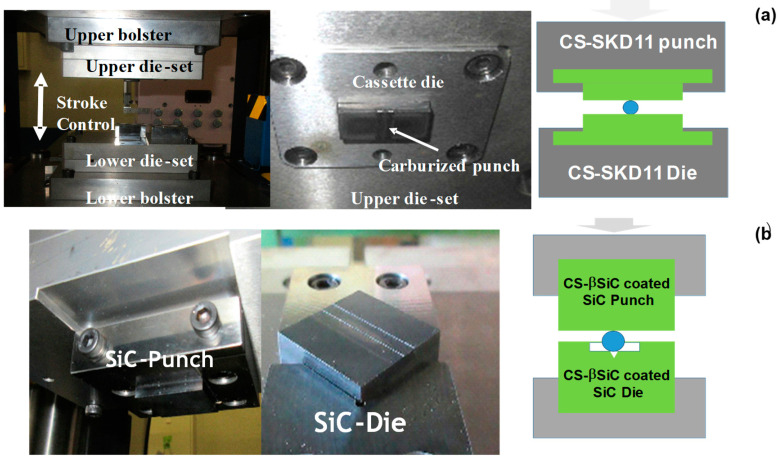
Two upsetting experimental setups. (**a**) Upsetting setup with the use of CS-SKD11 punch and die, and (**b**) upsetting setup with the use of CS-βSiC-coated SiC punch and die.

A CNC (computer numerical control)-stamping system (Hoden Seimitsu; Kanagawa, Japan) was employed for the upsetting experiments with the use of two different CS die pairs. The CS-SKD11 punch and die were fixed into the upper and lower cassette die sets, respectively, as depicted in Figure 5a. Those two die sets were cemented to the upper and lower bolsters of the CNC-stamping system with stroke control capability. The stroke was controlled by moving down the upper bolster to the specified position for reduction in thickness in work materials. The loading speed was constant at 10 mm/s. In a similar manner, the CS-βSiC-coated SiC punch and die were setup in the CNC stamper, as shown in Figure 5b. In both experiments, the load cell was embedded into the lower die set to monitor the variation in applied load by controlling the stroke.

### 2.4. Material and Mechanical Characterization

XRD (X-Ray Diffraction; D8, Bruker, Toyama, Japan) was used to detect the peak shift induced by carbon supersaturation. Optical microscopy (Shimazu, Co., Ltd., Kyoto, Japan) was first employed to understand the morphology of the contact interface on the punch. SEM-EDX (JOEL, Tokyo, Japan) was utilized for microstructure analysis on the contact interface. Raman spectroscopy (Nihon-Kogaku, Co., Ltd.; Tokyo, Japan) was utilized to analyze the binding state of iron, chromium, titanium, oxygen and carbon on the interface of the CS-SKD11 punch. It was also used to analyze the binding state of silicon, oxygen, titanium and carbon on the interface of the CS-βSiC-coated SiC punch.

## 3. Results

The carbon supersaturated dies are characterized by lattice expansion through XRD. The upsetting process was employed to describe the dry, cold forging of pure titanium by using the CS-SKD11 dies and the CS-βSiC-coated SiC dies.

### 3.1. Carbon Supersaturation to SKD11 and βSiC Coating Dies

**Figure 6:**
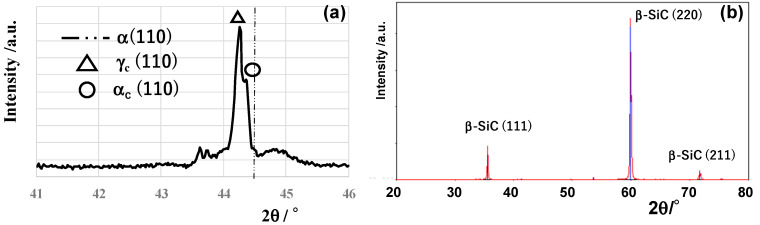
XRD diagram for CS-SKD11 die and CS-βSiC die. (**a**) XRD diagram of CS-SKD11 die in narrow 2θ range, and (**b**) XRD diagram of CS-βSiC die in wide 2θ range.

XRD was utilized in the normal scanning by 2θ–θ out-of-plain mode. In this operation, the increment of 2θ was constant by Δ(2θ) = 0.005°. In the XRD of the CS-SKD11 die, the original α-iron peak (110) in the bare SKD11 shifted to the lower 2θ side; e.g., the α-iron peak (110) at 2θ = 44.5° shifted to the peak at 2θ = 44.3° in Figure 6a in comparison to the XRD diagram for the original SKD11 in JCPDS- Cards No: 65-7296 and 88-2323. As reported in [21], this small peak shift proves the carbon supersaturation into SKD11 lattices. That is, the original bcc (body-center cubic) structure expands itself by carbon supersaturation. In addition to this peak shift, the expanded austenitic phase was also detected in Figure 6a.

Figure 6b compares the measured XRD diagram of SiC coating on the sintered SiC substrate and the bare βSiC and CS-βSiC coating dies. The normally CVD-coated βSiC is characterized by three peaks, e.g., βSiC (111) at 2θ = 36.05°, βSiC (200) at 2θ = 60.0°, and βSiC (331) at 2θ = 71.8°, respectively, with reference to [22]. This XRD reveals that the CVD-synthesized coating is β phase SiC and has a 3C-type cubic structure. Through the carbon supersaturation with co-deposition of βSiC onto the SiC block, these peaks in Figure 6b shifted themselves, e.g., βSiC (111) shifted to 2θ = 35.56°, and βSiC (220) shifted to 2θ = 59.88°. This carbon supersaturation is also characterized by the lattice expansion of 3C-structured grains. Differently from the CS-SKD11, this carbon supersaturation does not accompany two-phase structuring.

Later, the nanostructure of the CS layer in the SKD11 dies is discussed regarding its role in in situ lubrication based on the equivalence between nitrogen and carbon supersaturation processes. The intrinsic nature of the CS-βSiC layer is also reconsidered in the discussion.

### 3.2. Dry Forging of Pure Titanium Bar by CS-SKD11 Punch and Die

**Figure 7:**
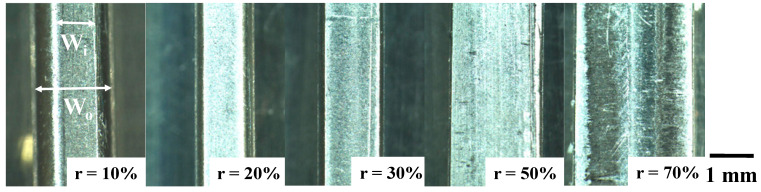
Variation in pure titanium wire with its width (W_o_) and its contact interface width (W_i_) with increasing reduction in thickness (r) until r = 70%.

A pure titanium wire with a diameter of 1.0 mm was prepared for a work in the dry cold upsetting experiment with high reduction in thickness (r) with the use of the CS-SKD11 punch and die. Figure 7 depicts the variation in upset specimens with incrementally increasing r; the raw wires were upset until r = 10%, 20%, 30%, 50% and 70%, respectively. In the normal upsetting process with a relatively high friction coefficient on the contact interface between the punch/die and the titanium work, r was limited to between 20 and 25% in reduction due to the risk of fracture by high tensile stress in the lateral direction [11]. As seen in Figure 7, both the wire width (W_o_) and the contact interface width (W_i_) increase monotonously with r, without any fracture or damage in work.

**Figure 8:**
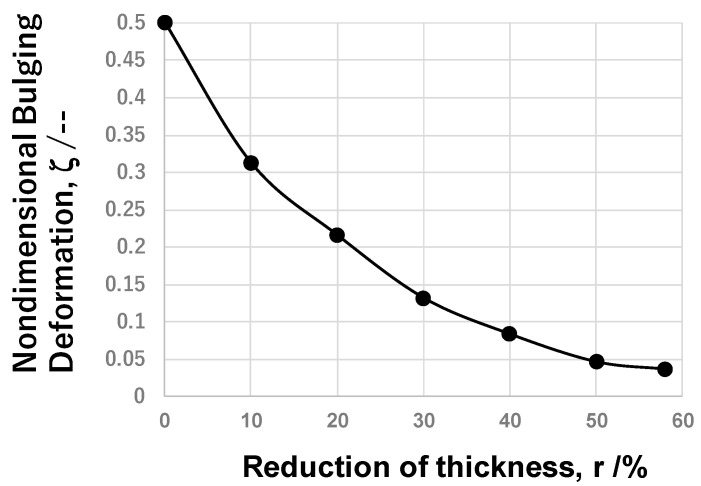
Variation in the nondimensional bulging deformation, ζ, with increasing reduction in thickness, r.

After [23], bulging deformation B_g_ (= (W_o_−W_i_)/2) is employed as a measure to describe the friction coefficient because B_g_ decreases monotonously with a reduction in the friction coefficient. Figure 8 depicts the variation in non-dimensional bulging deformation, ζ = (B_g_/W_o_), with r. ζ → 0 because the friction coefficient gradually decreases to zero with r. When r > 50%, ζ ~ 0, the titanium work plastically flows homogeneously along the contact interface and flattens by itself. This is completely different from the normal plastic flow of pure titanium, where the fresh surface of the titanium work easily comes into contact with the die at the risk of mass adhesion to the punch and die interfaces and increases in the friction coefficient. After the empirical estimate of the friction coefficient in [23], because W_o_ ~ W_i_, or, ζ → 0 at r > 30%, μ is estimated to be 0.05 to 0.1. In the literature, μ is lower bounded by μ = 0.2 when using a DLC-coated disc specimen against the pure titanium ball in BOD testing [8]. Even when the contact surface is textured and lubricated, μ is also lower bounded by μ = 0.18 in [9]. The above low friction coefficient with μ = from 0.05 to 0.1 implies that a lubricating tribofilm is formed on the CS-SKD11 die to titanium work during the upsetting process and that a low frictional interface condition is preserved in the successive upsetting operations.

This low frictional behavior on the contact interface reflects the work hardening behavior of titanium wires. Local deformations in the work materials were found to make a contribution to macroscopic work hardening or flow stress increase as suggested in [24]. Micro-hardness testing was employed to measure hardness mapping after the standard measurement methods in [25] and to describe the advancement of work hardening with increasing reduction in thickness.

**Figure 9:**
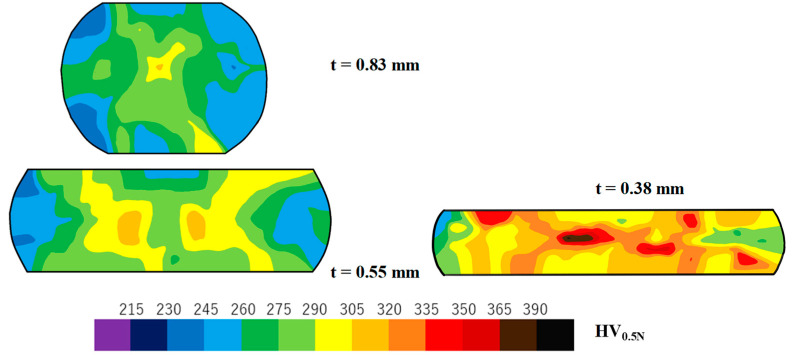
Variation in the cross-sections of titanium work and the hardness maps with increasing reduction (r) in thickness.

Figure 9 depicts the variation in work cross-sections and hardness maps with increasing r. Each hardness was measured pointwise with a distance of 0.1 mm. The measured data were edited and transformed into the colored contour diagram in Figure 9. Because the center part of the titanium wire is kept in compression during upsetting, higher hardness is measured irrespective of r. Except for these high hardness spots, the hardening process homogeneously advances with r across the whole cross-sections. No plastic localization was observed, even at r = 70%, which is different from the shear-band formation in [24] during the upsetting of wire- and disc-work specimens. This reveals that low friction on the contact interface results in significant suppression of work hardening by using the CS dies. It should be noticed that the upset titanium is shaped symmetrically with respect to the upper to lower halves and the right-hand to left-hand sides. This also implies that the titanium work flows homogeneously with low frictions on both interfaces of the CS punch and CS die to the work.

**Figure 10:**
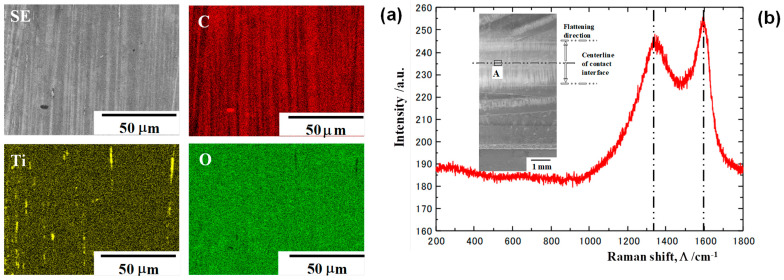
SEM-EDX analysis and Raman spectroscopy at the hot spots on the contact interface between the CS-SKD11 die and titanium work. (**a**) SEM image and element mapping at the center of the contact interface and (**b**) Raman spectrum at the same position.

SEM-EDX analysis was used to describe the microstructure and element mapping on these interfaces after continuously upsetting the titanium wires in twenty strokes by 70% in reduction, as shown in Figure 10a. In the SEM image at the center of the contact interface, white stripes were formed in a vertical direction from the centerline of the contact interface. On the Ti-mapping, a few linear deposits with a width of 2 μm were scarcely seen without correspondence to the white stripes in the SEM image. These white stripes were just in agreement with the carbon stripes in a vertical direction on the contact interface. That is, the carbon tribofilm with a fine stripe width and pitch of 1 μm, is formed in situ on the contact interface and aligned in the plastic flow directions during upsetting. Raman spectroscopy was also utilized to analyze the deposits on the interface. As shown in Figure 10b, no peaks were present in the wave number range from 100 cm^−1^ to 1000 cm^−1^. That is, no titanium oxides and carbides were synthesized on the contact interface; no debris particles were yielded on the contact interface. Only two significant peaks were detected at 1320 cm^−1^ and 1600 cm^−1^, respectively, corresponding to the Raman spectrum measured for the amorphous carbon in [26]. That is, an amorphous carbon tribofilm is formed in situ on the contact interface.

### 3.3. Upsetting by Using CS-βSiC Coated SiC Punch and Die

**Figure 11:**
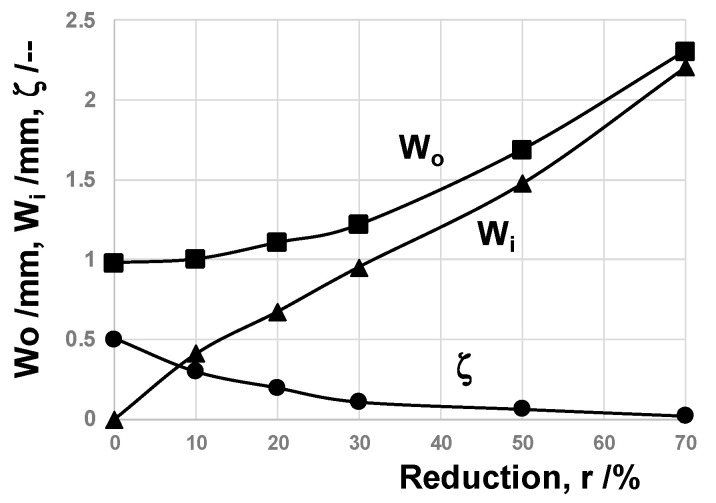
Variation in the work-bar width (W_o_), the contact interface width (Wi) and non-dimensional bulging displacement (ζ) with increasing r during upsetting.

The CS-βSiC-coated SiC punch and die were utilized for upsetting the pure titanium bar in dry and cold conditions. In a similar manner to the upsetting experiments with the use of CS SKD11 dies, the widths of the upset wire and contact interface were measured to describe the plastic work of titanium wires with increasing reduction in thickness (r). Non-dimensional bulging deformation, ζ, was also employed to describe flattening behavior. As shown in Figure 11, ζ = 0.5 at r = 0% because Wi = 0.0 mm and W_o_ = 1.0 mm, and Wi ~ Wo and ζ ~ 0 at r = 70%. As discussed for the upsetting by CS-SKD11 dies, the friction coefficient was empirically estimated to be 0.05 due to nearly zero bulging deformation for r = 70%. Irrespective of the substrate, a low frictional state is sustained during upsetting by CS-βSiC dies as well as CS-SKD11 dies.

**Figure 12:**
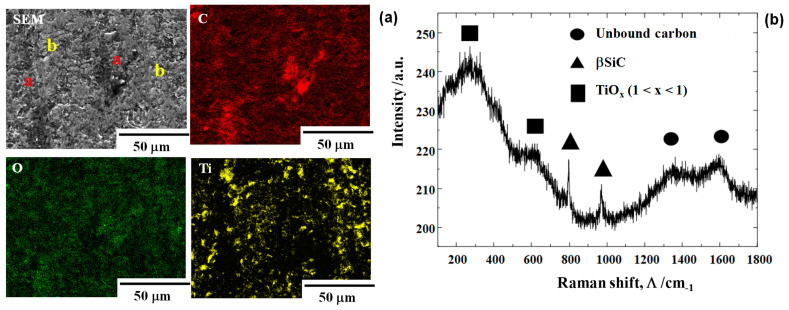
Microstructural analysis at the hot spots on the contact interface of the βSiC die and the titanium bar. (**a**) SEM image and element mapping by EDX and (**b**) Raman spectrum.

The contact interface between the βSiC coating and titanium work was analyzed using SEM-EDX. As seen in Figure 12a, two different zones are detected at the center region of the βSiC interface with the work. After carbon mapping, these “a” zones were identified as carbon agglomerates, formed on the interface. Whereas, the “b” zones were identified as titanium oxide debris fragments after Ti and O mapping, which are nearly coincident with each other. Fresh titanium oxidized at the hot spots on the interface. Notably, those debris fragments formed as a stripe in the direction of work plastic flow.

Raman spectroscopy was utilized to characterize the local material distribution at the center of the interface. As analyzed in Figure 12b, the titanium oxides of TiOx for 1 < x < 2 and free-carbon dots were formed on the interface of βSiC and the titanium work. Two broad D and G peaks were detected at Λ = 1320 cm^−1^ and 1600 cm^−1^, respectively. Two narrow peaks at Λ = 800 cm^−1^ and 950 cm^−1^, respectively, with weak intensities, reveal that the βSiC die surface coexists with a tribofilm. Compared to the high intensity peaks of βSiC at Λ = 800 cm^−1^ and 950 cm^−1^ just outside of the contact interface, their peak intensities become much smaller. This implies that the contact interface on the βSiC is mainly covered by the tribofilm including the carbon dots. In other peaks besides these two, a broad peak is only detected around Λ = 200 ~ 600 cm^−1^. If TiO_2_ were only present as a titanium oxide in the interface, narrow peaks could be detected at Λ = 400 cm^−1^ and 600 cm^−1^, respectively, after [27]. No detection of narrow peaks in Figure 12b implies that titanium oxide film consists of various binding states in the titanium—oxygen system from TiO to TiOx through the intermediate Magneli phase oxides [28]. In particular, other broad peaks at Λ = 150 and 250 cm^−1^ suggest that the intermediate titanium oxides or TiOx are formed together with TiO_2_ debris particles in the tribofilm. XPS (X-ray photoelectron spectroscopy) was utilized to investigate the binding energy of Ti—O in these oxides. Three Ti 3/2 peaks were detected for TiO, TiOx and TiO_2_. In these binding energies, 455 eV represented TiO, 457 eV corresponded to TiOx and 459 eV represented TiO_2_, respectively.

## 4. Discussion

Low friction and low work hardening of titanium work without adhesive wear or galling is common in upsetting processes using CS-SKD11 dies and CS-βSiC-coated SiC dies. This is a result of the in situ formation of a free-carbon tribofilm on the contact interface between dies and titanium work.

Let us discuss the mechanism in Figure 2 using EBSD (electron back-scattering diffraction) and STEM (scanning transmission electron microscopy) with reference to several studies in the literature. Theoretical study using the first-principles calculation demonstrated the equivalence between nitrogen supersaturation (NS) and carbon supersaturation (CS) processes involving irons [29]. The α-iron supercell lattice expands itself through occupation of its octahedral vacancy site by supersaturated interstitial solute. The carbon or nitrogen solute attracts its surrounding iron atoms to modify the electric structure of iron. These are common to two supersaturation processes. This equivalence is also experimentally validated in [30]. The peak shift of α-iron to lower 2θ by nitrogen and carbon supersaturation is observed by precise XRD analysis. The improvement of corrosion resistance by NS stainless steels is explained by the change in electric structure by solute supersaturation. Using these experimental results on the precise analyses on the NS stainless steels and tool steels, let us describe the role of interstitial-induced nanostructure to drive the isolation and diffusion of free-carbon atoms from the CS dies. Then, the effect of carbon interstitial supersaturation on microstructure evolution is explained by microstructure analysis of the nitrogen supersaturated dies.

**Figure 13:**
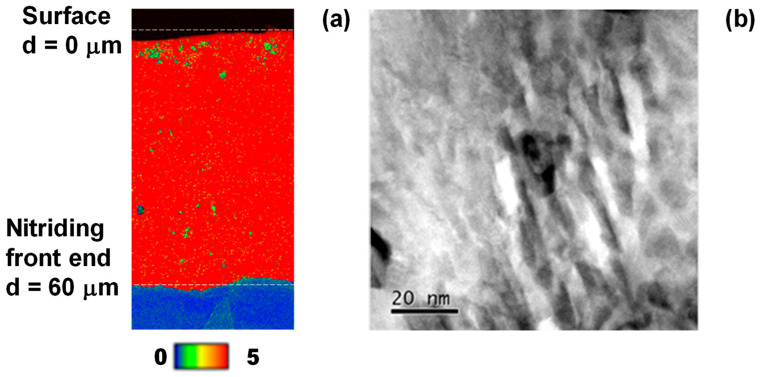
EBSD and STEM analyses on the cross-section of NS die from its surface to the depth across the nitriding front end. (**a**) KAM distribution or plastic strain distribution, and (**b**) LAADF image on the cross-check nanostructure, induced by the slip-line field.

EBSD and STEM were utilized to analyze crystallographic misorientation in the cross-section of NS die. Figure 13a depicts the KAM (Kernel angle misorientation) profile at the NS layer toward the nitriding front end. The whole cross-section of the NS layer is covered by a higher angle misorientation than 5°. This implies that the NS die microstructure is subjected to high plastic straining everywhere. After [31], the highly plastically strained microstructure is induced by slip-line field formation along the crystallographic orientation of (111). Figure 13b shows the LAADF image of the NS layer by STEM; the skewed slip lines are densely observed in the image. This proves that interstitial supersaturation accompanies high plastic straining to modify the original microstructure to have a fine diffusion network of interstitial atoms. That is, the carbon solutes can diffuse themselves through the cluster boundary network.

**Figure 14:**
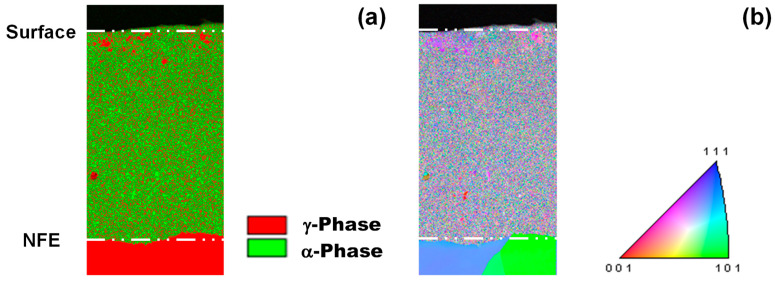
Phase mapping and inverse pole figure of NS layer analyzed by EBSD. (**a**) Phase mapping, and (**b**) inverse pole figure map.

As seen in Figure 6a, α- and γ- broad peaks are detected by XRD as a shifted peak by NS and CS. NS and CS layers have fine clustering and a two-phase structure. EBSD is utilized to describe this two-phase nanostructure by using the phase map and inverse pole figure (IPF) map. As shown in Figure 14, each nanograin with γ-phase is neighboring another nanograin with α-phase. This implies that each cluster in Figure 13b corresponds to a nanograin in Figure 14a,b. That is, NS and CS modify the original microstructure of the dies to a clustered nanostructure with different crystallographic structures. The cluster with rich interstitial solutes corresponds to the γ-phase nanograin, whereas the cluster with poor interstitial solutes corresponds to the α-phase nanograin [32]. These two different nanograins are neighboring each other. Under the externally applied stress gradient, the interstitial solutes in clusters make a jumping diffusion from the original vacancy site to the other and further diffuse by themselves through the cluster boundary to the contact interface after [33].

This mechanism is true to the carbon solute isolation and diffusion in the CS-SKD11 dies. Before metal forming, the supersaturated carbon solutes occupy each vacancy site both in the carbon-rich and carbon-poor clusters. The cluster boundary works as a stable zone for carbon solute to jump up from the original site in each cluster under the stress gradient field. Then, each carbon solute diffuses through the cluster boundaries to the hot spots on the contact interface and forms a free-carbon tribofilm in situ.

Let us consider the galling free forging of titanium by CS-βSiC from two aspects. First, the electric structure differs between covalent βSiC with a 3C structure and metallic titanium work. After the first-principles calculation in [34], two matters with nearly the same density of state (DOS) in their local electron structure, are easy to adhere to each other; whereas, two with significant differences in DOS have less bound interface. Dense β-SiC coating punch and die have a covalent bonding state in the cubic structure, whereas titanium is a typical transition metal that exhibits metallic bonding characterized by free electron pairs. No detection of metallic titanium by the EDX analysis in Figure 12 reveals that DOS on the βSiC surface still remains completely separate from that on the metallic titanium. Detection of titanium oxide on the βSiC die surface also proves that flakes of oxide layer on the work wire are mechanically splashed into the air and are deposited onto the slightly rough surface of the βSiC die surface.

Secondly, the free-carbon agglomerates appearing as a dot on the contact interface between βSiC and titanium work in Figure 12 must be considered. XRD analysis reveals that lattice expansion by CS-βSiC is detected by the peak shift in the lower 2θ. Although each cell in βSiC elastically strains by itself, the neighboring cells distort with much less plastic strain. Most of the impinged carbon solutes occupy the vacancy site near the zone boundary or the defective zones near the grain boundaries. Under the stress gradient, these free-carbon solutes isolate from those sites to zone and grain boundaries and diffuse through these paths to the hot spots on the contact interface in a similar manner to the in situ solid lubrication process observed in CS-SKD11 dies. In fact, the free-carbon agglomerate size in Figure 12a is equivalent to the grain size.

## 5. Conclusions

Carbon supersaturated SKD11 dies and βSiC-coated SiC dies were prepared for dry, cold forging experiments. Pure titanium wires were upset in a single shot by a 70% reduction in thickness with low friction and low galling wear. The precise SEM-EDX and Raman spectroscopy analyses proved that an amorphous free-carbon tribofilm was formed in situ on the true contact interface between the CS dies and the titanium work. This low friction and low galling wear on the contact interface suppressed the work hardening in the titanium work. These features in the metal-forming tribology are favored for high quality manufacturing of titanium medical tools and parts.

EBSD and STEM analyses showed that the nanostructures of CS-SKD11 dies accommodate the diffusion network for carbon solutes. When the stress gradient is applied during metal forming, these solutes diffuse and agglomerate themselves as a tribofilm on the contact interface. This in situ solid lubrication by free-carbon dots characterizes the dry, cold upsetting behavior of the CS-βSiC coating die. The carbon solutes near the zone and grain boundaries isolate from βSiC grains, diffuse to the interface through these boundary networks and agglomerate as free-carbon dots with nearly the same size as βSiC grains.

Galling-free metal forming using CS tools is useful in forging, press-forging and in the fine blanking steps used to achieve near-net shaping of difficult-to-form work materials even with a severe plastic flow of works. The die material design is essential to control the constituent alloying elements with different affinities to the interstitial solutes in the supersaturation process and to facilitate the in situ formation of tribofilms on the hot spots of dies in a feasible way in each metal forming step.

## Figures and Tables

**Table 1:** Plasma carburizing conditions of the heat-treated SKD11.

Parameter	Temperature	Pressure	Duration	RF-Voltage
**Presputtering**	673 K	70 Pa	1.8 ks	-
**Nitriding**	673 K	70 Pa	14.4 ks	250 V
**Parameter**	**DC-Bias**	**Argon** **flow rate**	**Hydrogen** **flow rate**	**Methane** **flow rate**
**Presputtering**	−500 V	160 mL/min	20 mL/min	-
**Nitriding**	−500 V	160 mL/min	20 mL/min	20 mL/min

## Data Availability

The data presented in this study are available on request from the corresponding author.
